# A convenient method for the construction of triazole-bonded chalcone derivatives from acetophenone: Synthesis and free radical scavenging investigation

**DOI:** 10.1016/j.mex.2023.102322

**Published:** 2023-08-07

**Authors:** Atta Ullah, Nur Rohman, Bayu Ardiansah, Antonius Herry Cahyana, Abdulrahman A. Almehizia

**Affiliations:** a Department of Chemistry, Faculty of Mathematics and Natural Sciences, Universitas Indonesia, Depok 16424, Indonesia; b Department of Pharmaceutical Chemistry, College of Pharmacy, King Saud University, Riyadh 11451, Saudi Arabia

**Keywords:** 1,2,3-triazole, Chalcone, Copper-mediated azide-alkyne cyclization, Radical scavenging, Synthesis of triazole-bonded chalcone derivatives via three steps reaction: aldol condensation, propargylation, and then copper-mediated azide-alkyne cyclization

## Abstract

The substituted 1,2,3-triazole core is prevalent in numerous commercially available drugs utilized for a wide range of clinical applications. Simultaneously, chalcone represents a privileged framework discovered in natural products exhibiting intriguing bioactivities. In this study, we synthesized triazole-bonded chalcone compounds (**4ax**-**4by**), starting from a simple aromatic ketone, acetophenone, which underwent aldol condensation to give hydroxychalcone intermediate. In the second step, the hydroxyl group of chalcone compound was adducted with propargyl moiety through propargylation reaction. Then, the propargylated products underwent smooth copper-mediated azide-alkyne cyclization to give the triazole-bonded chalcones as the final products. They were characterized by IR, NMR and HRMS, and evaluated their radical scavenging activity against 2,2-diphenyl-1-picrylhydrazyl (DPPH). Among the tested products, compound **4by** was denoted as the most potent derivative which can inhibit DPPH radical in 91.62 ± 0.10% at 500 ppm.

• Acetophenone as a simple ketone was modified to triazole-bonded chalcones.
• Modification was performed through three steps reaction.
• Final products exhibited free radical scavenging activity.

## Graphical abstract

**Figure:**
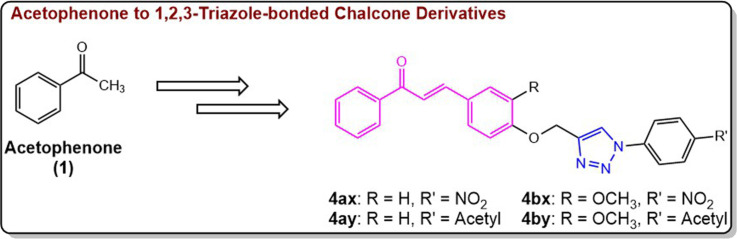

Specifications Table

**Table:** 

Subject area:	Chemistry
More specific subject area:	Organic Chemistry
Name of your method:	Synthesis of triazole-bonded chalcone derivatives via three steps reaction: aldol condensation, propargylation, and then copper-mediated azide-alkyne cyclization
Name and reference of original method:	Synthesis of triazole derivatives Bioorg. Med. Chem. Lett., 2020, 30, 127434.
Resource availability:	The investigation was conducted in the Laboratory of Organic and Biochemistry, Department of Chemistry, Faculty of Mathematics and Natural Sciences, Universitas Indonesia, Depok. The reagents and chemicals used were purchased from commercial suppliers such as Merck, Sigma-Aldrich, and PT. Smart Lab Indonesia. The instruments used were FTIR, ^1^H and ^13^C NMR, and HRMS.

## Method details

Chalcones are excellent compounds having α, β-unsaturated ketone functionality between the two aromatic rings. They are flavonoids precursors and have been extensively researched as essential structural elements in medication development [1]. Many pharmacological properties of chalcone and its derivatives have been reported in recent studies, including anticonvulsant, anti-inflammatory, antimalarial, anticancer, antioxidant, antibacterial, anti-proliferative, and xanthine oxidase inhibitory effects [2,3]. Heterocycles have been crucial in drug discovery. The structural motif 1,2,3-triazole is a neoclassical bioisostere of amide, which is a frequently used functional group in approved medications [4]. In general, 1,2,3-triazole has a number of advantages in drug development [5]. For example, triazoles have two H-bond acceptors and can interact with biomolecular targets through H-bonds, π-π stacking, and dipole interactions. In recent studies, organic compounds bearing 1,2,3-triazole have shown good activity as antioxidant [6,7], and other biological properties like anticancer [8] and antidiabetic [9]. With thie above advantages, we synthesized the triazole-bonded chalcone compounds as described as follows.

**Scheme 1:**
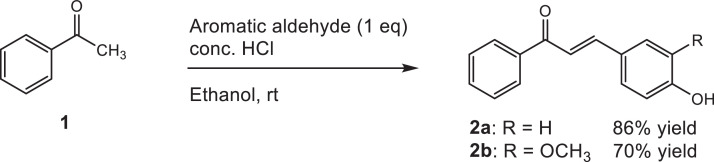
Aldol condensation to produce hydroxychalcone.

Our method is aimed to synthesize 1,2,3-triazole-bonded chalcones from simple aromatic ketone. To do it, acetophenone (**1**) was used as a starting material. Acetophenone (20 mmol, 2.33 mL), aromatic aldehyde (20 mmol), and ethanol (2 mL) were placed in a 50 mL round bottom flask. The reaction mixture was cooled down to 0°C and stirred for 5 min, then concentrated hydrochloric acid (5 mL) was added. The resulting mixture was stirred for 24 h at room temperature. After that, cold water (15 mL) and glacial acetic acid (15 mL) were added to the flask. The formed solid was filtered, washed with cold water, and then dried to give crude material, which was purified by silica gel column chromatography (hexane / ethyl acetate = 10 / 1 to 1 / 1) to give the corresponding hydroxychalcone **2a** ((*E*)-3-(4-hydroxyphenyl)-1-phenylprop-2-en-1-one) or **2b** ((*E*)-3-(4-hydroxy-3-methoxyphenyl)-1-phenylprop-2-en-1-one), respectively. Aldol condensation for the synthesis of chalcones (similar compounds to our research) were already performed by Sharma *et al.* (2014) in basic media using sodium hydroxide methanolic-aqueous solution [10]. However, in our experiments, performing aldol condensation in such condition was difficult when the substrate contains phenolic group, the product was obtained in low yield. Fortunately, under acidic condition, compounds **2a** and **2b** were obtained in good yield (Scheme 1).

**Scheme 2:**
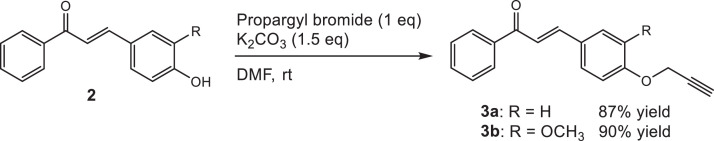
Propargylation of hydroxychalcone.

The products from the aldol condensation were used in the second step. Propargylation reaction is a process to introduce propargyl moiety to a chemical structure [11]. For propargylation of phenolic compounds, potassium carbonate is often used as deprotonating agent [12]. Propargylation reaction of hydroxchalcone was performed by the following conditions (Scheme 2). In a 30 mL round bottom flask containing chalcone (5.0 mmol) in dimethylformamide (10 mL), potassium carbonate (10.0 mmol, 1.39 g) was added, and the mixture was stirred at room temperature for 1 h. A solution of propargyl bromide 80 wt.% in toluene (10.0 mmol, 1.12 mL) was added to the mixture, and the resulting solution was stirred at room temperature for 24 h. After that, the mixture was poured into cold water. The obtained solid was filtered and washed with cold water to afford propargylated chalcone **3a** ((*E*)-1-phenyl-3-(4-(prop-2-yn-1-yloxy)phenyl)prop-2-en-1-one) or **3b** (*(E)-*3-(3-methoxy-4-(prop-2-yn-1-yloxy)phenyl)-1-phenylprop-2-en-1-one), respectively.

**Scheme 3:**
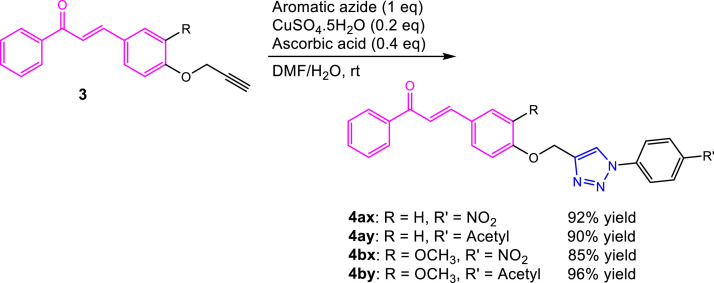
Copper-mediated azide-alkyne [3+2] cyclization reaction.

Final step in our work is copper-mediated azide-alkyne [3+2] cyclization (Scheme 3). The typical reactions involve alkyne (propargylated compound, for example) and organic azide via 1,3-dipolar cycloaddition. Heating those substrates in the absence of catalyst will give a mixture of 1,4- and 1,5-disubstituted triazoles [13]. By using copper(I), the cycloaddition proceeds regioselectively to form 1,4-regioisomer [14,15]. In this experiment, we utilized *in situ* prepared Cu(I) from the redox reaction between copper(II) sulfate pentahydrate and ascorbic acid. In a 10 mL round bottom flask, a mixture containing propargylated chalcone (0.5 mmol), aromatic azide (0.5 mmol), ascorbic acid (0.20 mmol, 35.2 mg), and copper(II) sulfate pentahydrate (0.10 mmol, 25.0 mg) in dimethylformamide/water (1:1, 2.5 mL DMF and 2.5 mL water) was stirred at room temperature. After the completion of the reaction, the mixture was poured into ice cold water. The obtained solid was filtered, washed with excess water, and dried to afford triazole-bonded chalcone derivatives (**4ax**-**4by**).

The synthesized derivatives of 1,2,3-triazole-bonded chalcone derivatives were confirmed by Infrared (IR), Nuclear Magnetic Resonance (^1^H- and ^13^C-NMR), and High Resolution Mass Spectrometry (HRMS). The presence of conjugated carbonyl stretch in all compounds was confirmed at 1651–1654 cm^−1^. For acetyl derivatives of triazole-chalcone (compound **4ay** and **4by**), additional carbonyl stretches which are conjugated with phenyl ring was observed at 1679–1681 cm^−1^. From ^1^H-NMR, singlet signal at around 9.12–9.19 ppm indicated the presence of vinylic proton in triazole ring. The methoxy group in compound **4bx** and **4by** was indicated at peak 3.86 and 3.88 ppm, respectively. Meanwhile, methyl ketone proton in compound **4ay** and **4by** was confirmed at 2.65 ppm. In ^13^C-NMR, carbonyl of enone moiety was confirmed at 189 ppm. Meanwhile, carbonyl of acetyl group was observed at 197 ppm (compound **4ay** and **4by**) Methylene carbon next to triazole ring was indicated by peak at range 55–61 ppm. Finally, the findings of HRMS of synthesized derivatives were found to be very close to the theoretical values. Mass spectra of the synthesized derivatives reflected the characteristic [M+H]^+^ ion peaks.

In compared to other approaches, DPPH radical scavenging is a prominent methodology for measuring antioxidant activity in a very short period of time [16]. Using an ethanolic solution of the stable free radical DPPH, the antioxidant ability of the synthetic compounds (**4ax**–**4by**) was evaluated (Table 1). The sample solution (500 ppm, 2 mL) was added to the DPPH solution (100 ppm, 1 mL) in 2 mL of methanol. After homogenization, the solution was incubated for 30 min in dark condition. Absorption was read at 517 nm by UV-Vis spectrophotometer and compared with the blank. Based on evaluation, all final products, including compound **2a** and **2b** as their starting materials, showed radical scavenging activity. Compound **4by** which contain methoxy and acetyl group was denoted as the most potent agent with inhibition capacity of 91.62 ± 0.10%. It may be attributed from the electronic effect of methoxy and acetyl group that can enhance the scavenging capacity.

**Table 1:** Inhibition of DPPH by the synthesized products at 500 ppm.

Compound	% Inhibition
**2a**	82.37 ± 0.10
**2b**	89.01 ± 0.10
**4ax**	43.40 ± 1.25
**4ay**	73.59 ± 0.25
**4bx**	81.33 ± 0.33
**4by**	91.62 ± 0.10
**Ascorbic Acid**	98.66 ± 0.06 (at 25 ppm)

## CRediT authorship contribution statement

**Atta Ullah:** Investigation, Validation, Data curation. **Nur Rohman:** Investigation, Validation, Data curation. **Bayu Ardiansah:** Conceptualization, Methodology, Supervision, Writing – original draft. **Antonius Herry Cahyana:** Methodology, Supervision. **Abdulrahman A. Almehizia:** Supervision, Writing – review & editing.

## Declaration of Competing Interest

The authors declare that they have no known competing financial interests or personal relationships that could have appeared to influence the work reported in this paper.

## Data Availability

Data will be made available on request.

## Appendix Supplementary materials

Image, application 1
