# Supplementary material for: A convenient method for the construction of triazole-bonded chalcone derivatives from acetophenone: Synthesis and free radical scavenging investigation
Source: MethodsX. 2023 Aug 7;11:102322. doi: 10.1016/j.mex.2023.102322 (PMC10440577; doi:10.1016/j.mex.2023.102322)

**Supplementary material *and/or* additional information**

The supplementary material below contains analytical data and spectral data (IR, HRMS, ^1^H-, and ^13^C-NMR) of the synthesized compounds.

*General information:*

Organic azides have the potential to be explosive and dangerous. All manipulations were carried out carefully behind a safety shield in a hood to prevent explosions, even though we have never experienced such serious incidents during our studies. The use of a plastic spatula is necessary for transferring sodium azide. Special attention should be given to azido compounds with a (C+O)/N ratio of less than 3, according to Smith's ratio. All reagents were purchased from Sigma-Aldrich and Merck. Anhydrous solvents such as methanol and ethanol were obtained from Merck and PT. SMARTLAB Indonesia. Distilled water was used as the reaction solvent, for quenching reactions, and during separation procedures. At the Integrated Laboratory and Research Center (ILRC) of Universitas Indonesia, ^1^H and ^13^C NMR spectra were recorded using a Bruker Avance Neo 500 MHz spectrometer. Deuterated dimethyl sulfoxide (DMSO-d6) was used as the internal standard for chemical shifts, which are reported in ppm (^1^H: 2.51, ^13^C: 39.53 ppm). In some cases, deuterated chloroform (CDCl_3_) was used (^1^H: 7.26, ^13^C: 77.00 ppm). The abbreviations used are as follows: s (singlet), d (doublet), t (triplet), q (quartet), and m (multiplet). Melting points were measured using an Electrothermal IA9100. Infrared spectra were obtained using a Shimadzu IRPrestige-21 FTIR spectrometer. High-Resolution Mass Spectra (HRMS) were acquired using the Xevo G2-XS QTof instrument (Waters, USA) with ESI-TOF detection mode at the Forensic Laboratory Centre of the Indonesia National Police. The progress of the reaction was monitored using silica gel thin-layer chromatography (TLC) (Merck TLC Silica Gel 60 F254) and visualized under a UV lamp. Column chromatography was performed using Merck silica gel 60.

*Analytical and spectral data:*

***(E)-3-(4-hydroxyphenyl)-1-phenylprop-2-en-1-one (2a****)*

Yellow solid; R*_f_* value 0.37 (hexane / ethyl acetate = 3 / 1); m.p. 64–66 °C; IR (KBr, disc) ν_max_ 3220, 3038, 1660, 1599, 1575, 1510, 1343, 1294, 1216, 1172, 1033, 984 cm^-1^; ^1^H NMR (500 MHz, DMSO-*d*6) δ 10.11 (s, 1H), 8.11 (dd, 2H, *J* = 8.1, 1.1 Hz), 7.73 (d, 2H, *J* = 8.7 Hz), 7.70 (d, 2H, *J* = 4.4 Hz), 7.65 (tt, 1H, *J* = 7.3, 1.9 Hz), 7.55 (t, 2H, *J* = 7.3 Hz), 6.84 (d, 2H, *J* = 8.7 Hz); ^13^C NMR (126 MHz, DMSO-*d*6) δ 189.1, 160.2, 144.6, 138.0, 132.9, 131.1, 128.8, 128.4, 125.8, 118.5, 115.9; HRMS (ESI-TOF) calcd for C_15_H_13_O_2_ [M+H]^+^ 225.0916, found 225.0919.


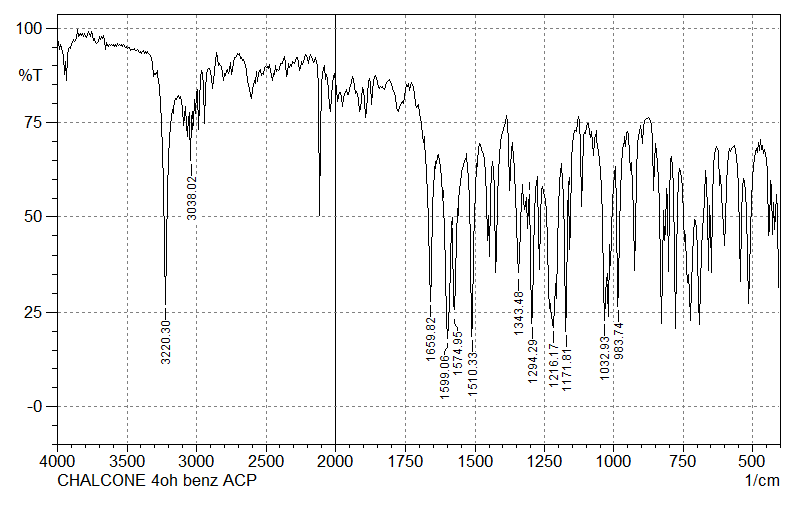


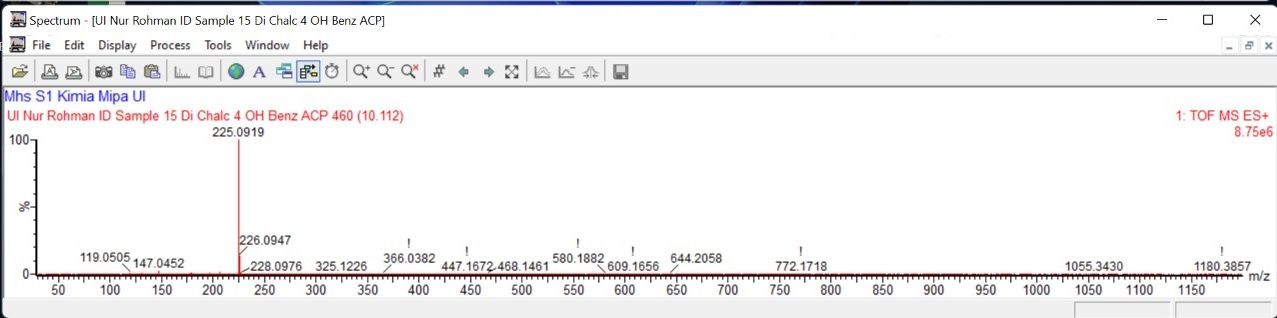


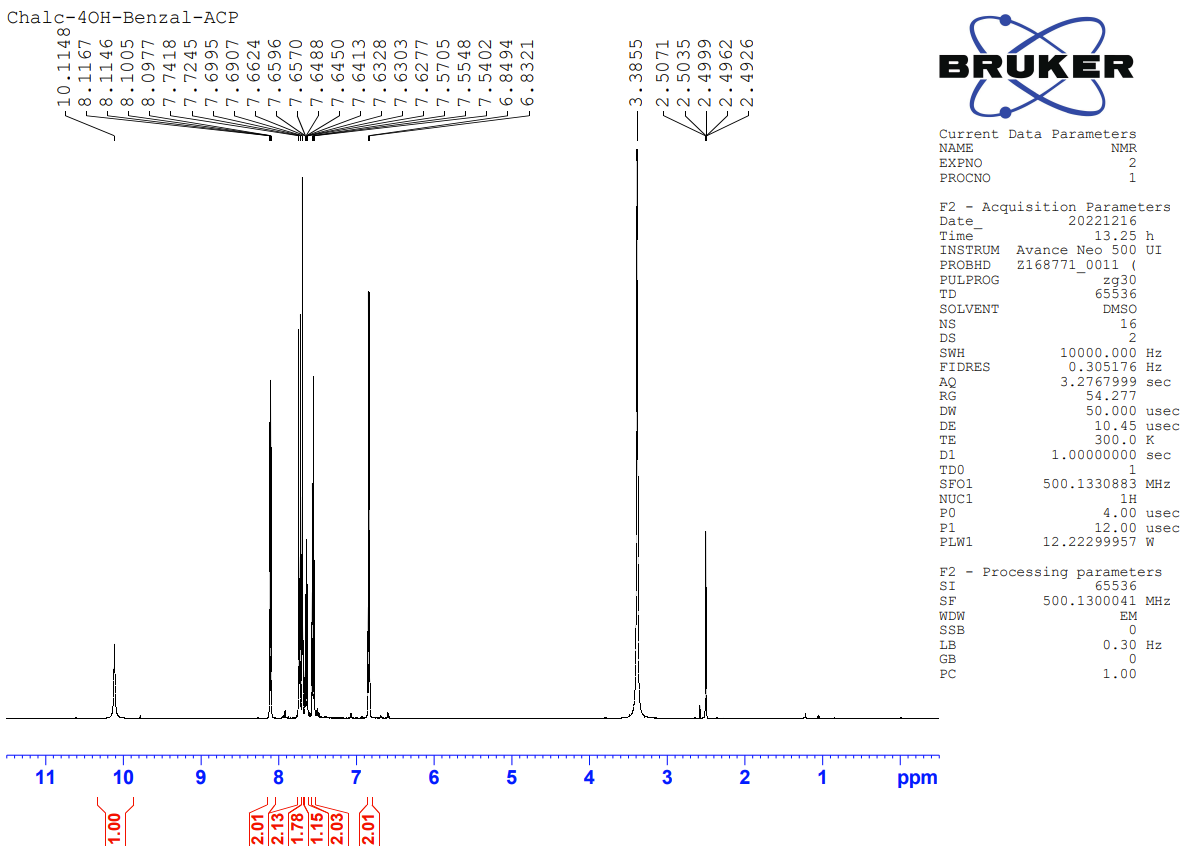


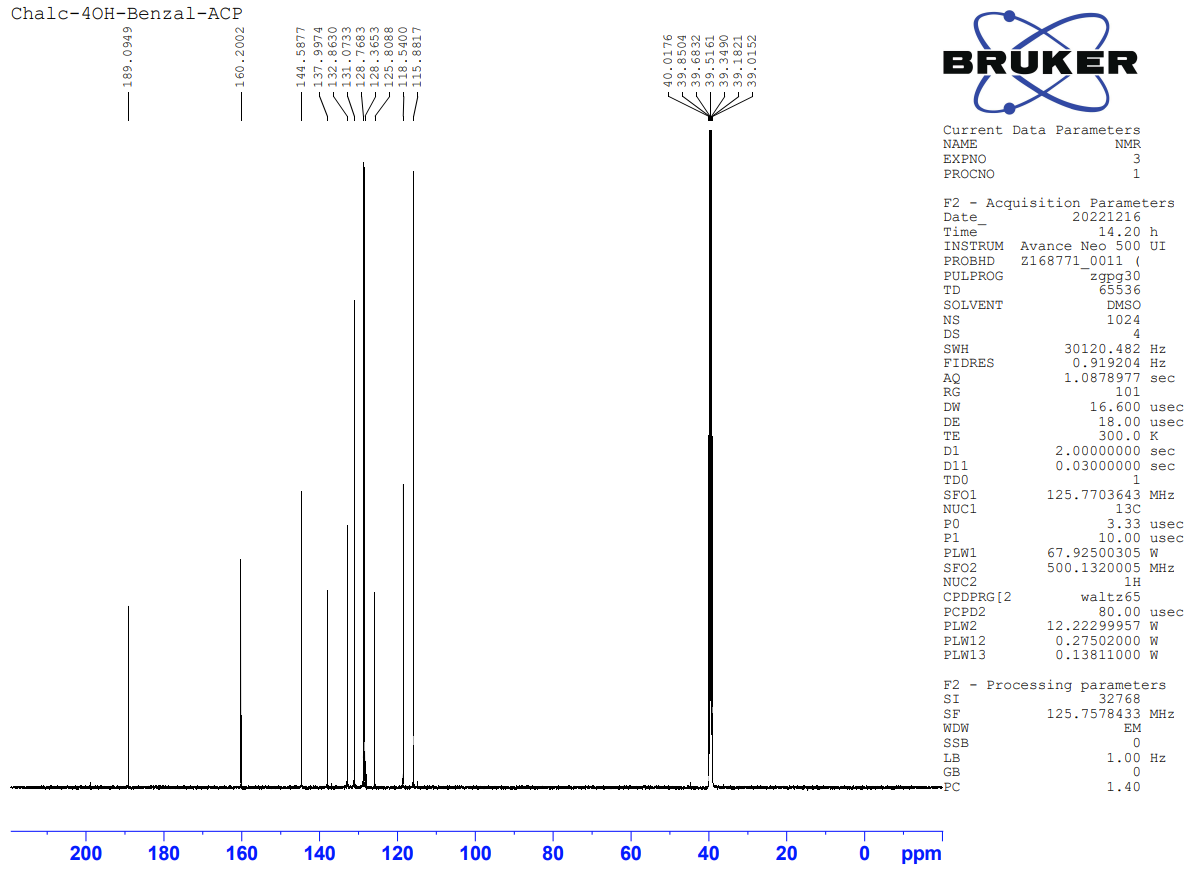


***(E)-3-(4-hydroxy-3-methoxyphenyl)-1-phenylprop-2-en-1-one (2b):***

Yellow solid; R*_f_* value 0.37 (hexane / ethyl acetate = 3 / 1); m.p. 72–74 °C; IR (KBr, disc) ν_max_ 3325, 3062, 2960, 2873, 1727, 1657, 1587, 1527, 1455, 1371, 1275, 1126, 1040, 982 cm^-1^; ^1^H NMR (500 MHz, CDCl_3_) δ 8.00 (dd, 2H, *J* = 8.2, 1.5 Hz), 7.75 (d, 1H, *J* = 15.6 Hz), 7.70 (dd, 1H, *J* = 5.7, 3.3 Hz), 7.56 (tt, 1H, *J* = 7.35, 2.1, 1.3 Hz), 7.52 (dd, 1H, *J* = 5.7, 3.3 Hz), 7.49 (d, 1H, *J* = 7.8 Hz), 7.37 (d, 1H, 15.6 Hz), 7.20 (dd, 1H, *J* = 8.3, 1.9 Hz), 7.12 (d, 1H, *J* = 1.9 Hz), 6.95 (d, 1H, *J* = 9.6 Hz), 3.93 (s, 3H); ^13^C NMR (126 MHz, CDCl_3_) δ 190.7, 148.4, 146.8, 145.3, 138.4, 132.5, 128.7, 128.5, 128.4, 123.3, 119.7, 114.9, 110.0, 55.9; HRMS (ESI-TOF) calcd for C_16_H_15_O_3_ [M+H]^+^ 255.1021, found 255.1022.


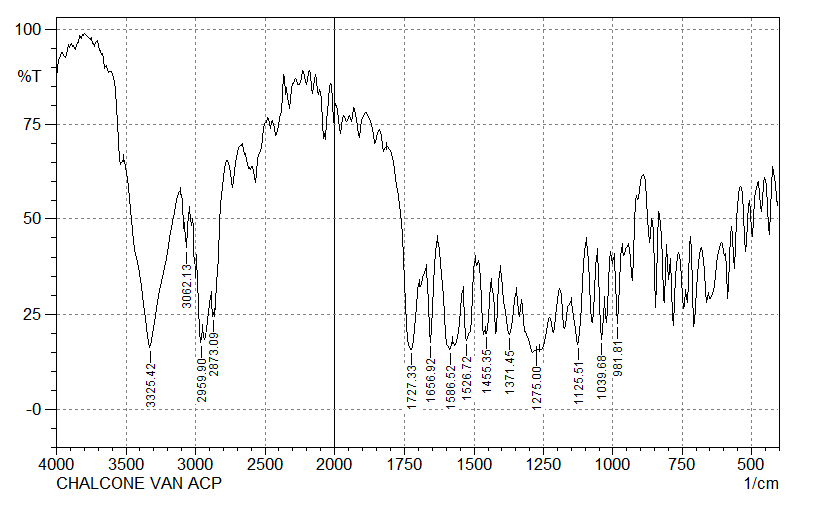


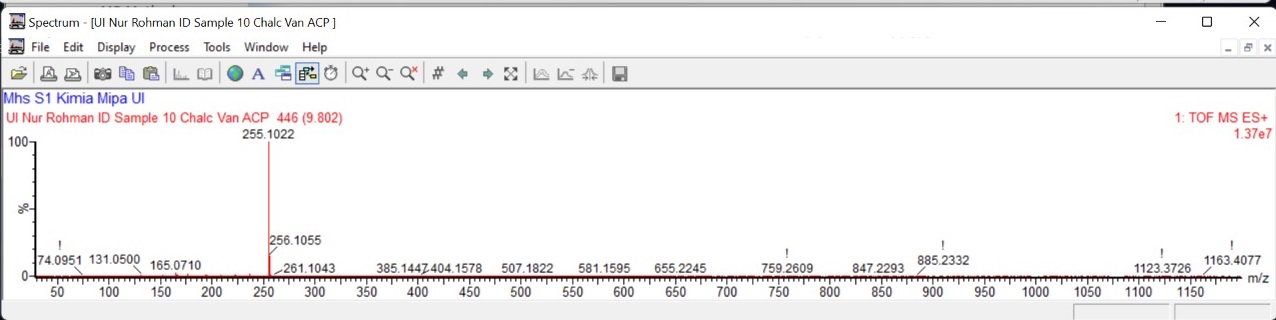


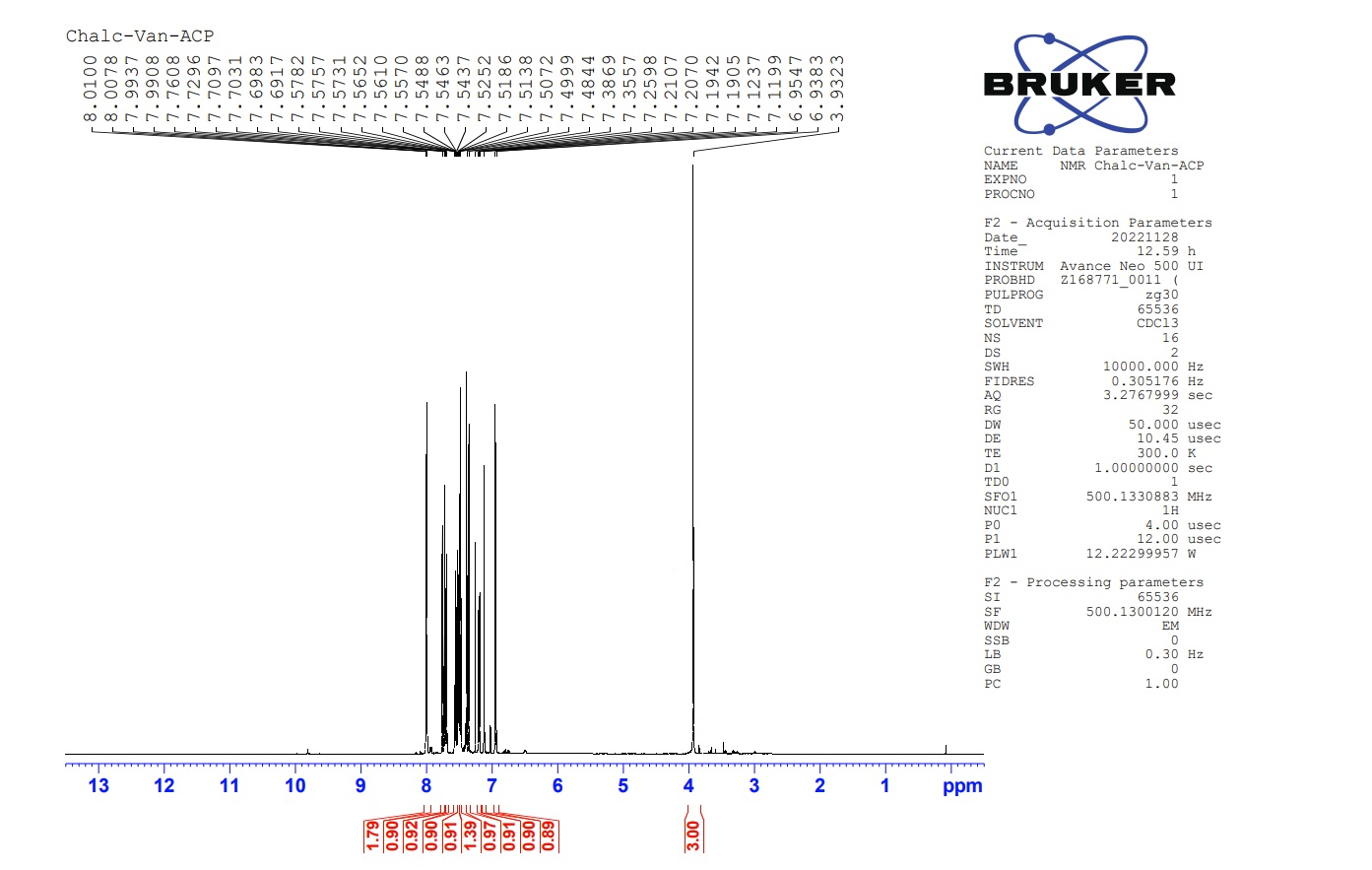


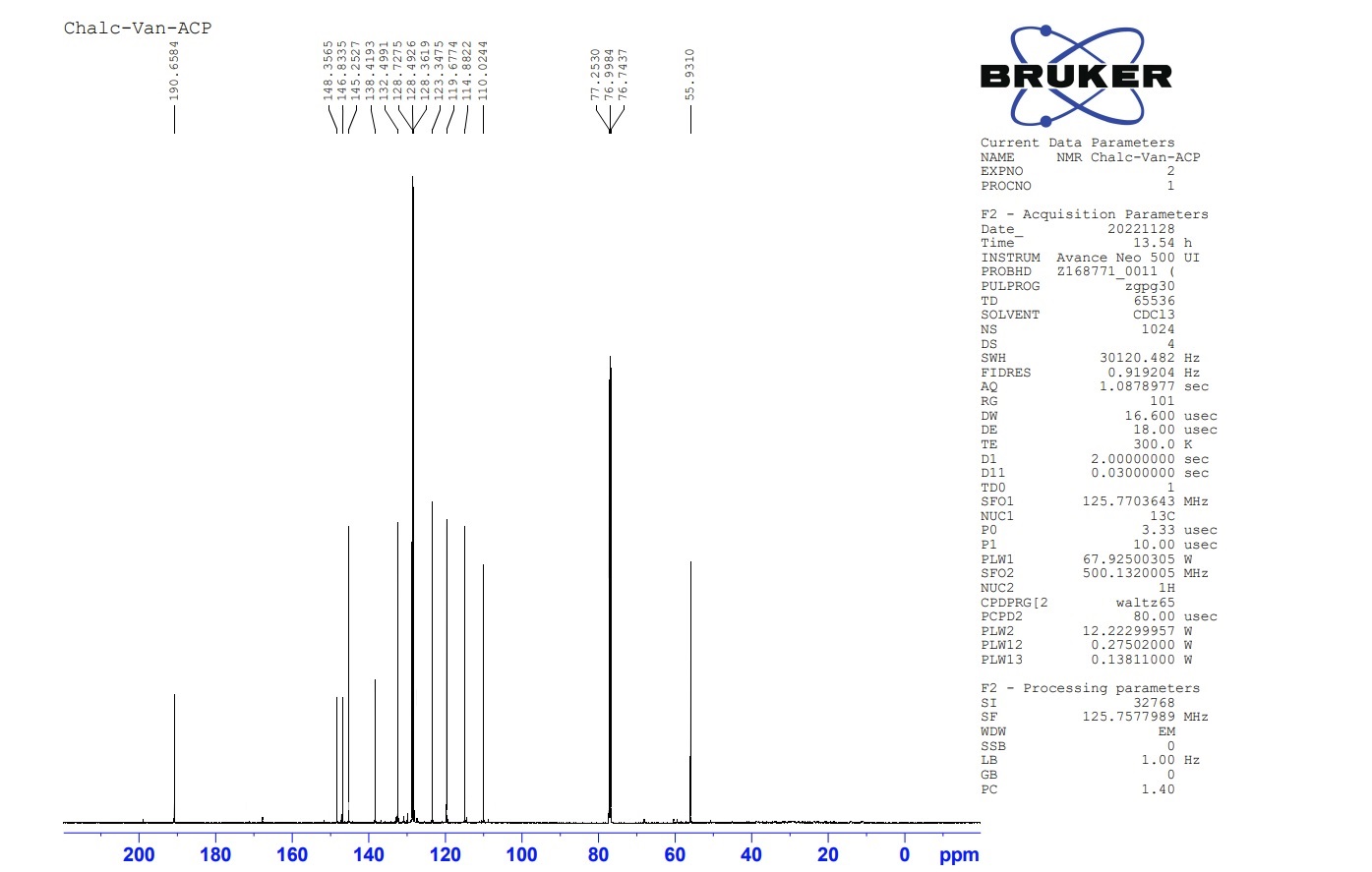


***(E)-1-phenyl-3-(4-(prop-2-yn-1-yloxy)phenyl)prop-2-en-1-one (3a):***

Off-white solid. R*_f_* value 0.75 (hexane / ethyl acetate = 3 / 1); mp. 69–70 °C; IR (KBr, disc) ν_max_ 3220, 3038, 2110, 1660, 1598, 1510, 1424, 1343, 1294, 1216, 1172, 1033, 984 cm^-1^; ^1^H NMR (500 MHz, DMSO-*d*6) δ 8.13 (dd, 2H, *J* = 8.5, 1.4 Hz), 7.87 (d, 2H, *J* = 8.8 Hz), 7.82 (d, 1H, *J* = 15.6 Hz), 7.72 (d, 1H, *J* = 15.6 Hz), 7.66 (tt, 1H, *J* = 7.4, 1.8 Hz), 7.57 (t, 2H, *J* = 7.9 Hz), 7.07 (d, 2H, *J* = 8.8 Hz), 4.89 (d, 2H, *J* = 2.4 Hz), 3.61 (t, 1H, *J* = 2.4 Hz); ^13^C NMR (126 MHz, DMSO-*d*6) δ 189.1, 159.2, 143.8, 137.8, 133.0, 130.7, 128.8, 128.4, 128.0, 120.0, 115.3, 78.9, 78.6, 55.6; HRMS (ESI-TOF) calcd for C_18_H_15_O_2_ [M+H]^+^ 263.1072, found 263.1088.


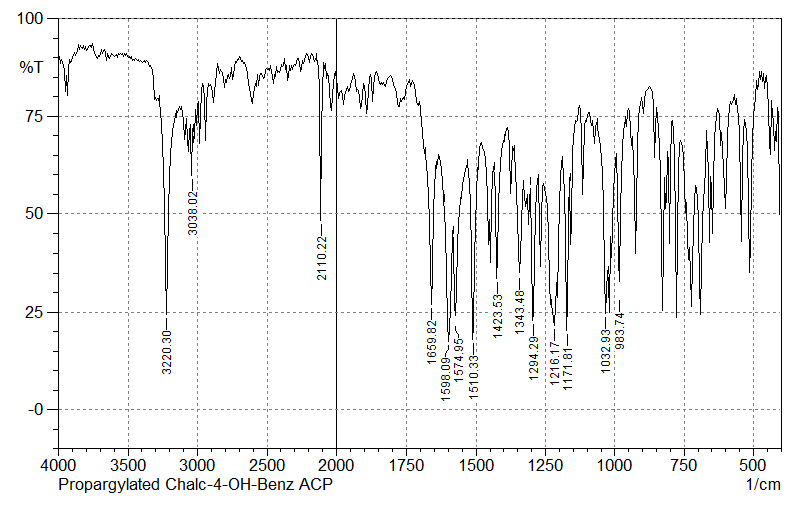


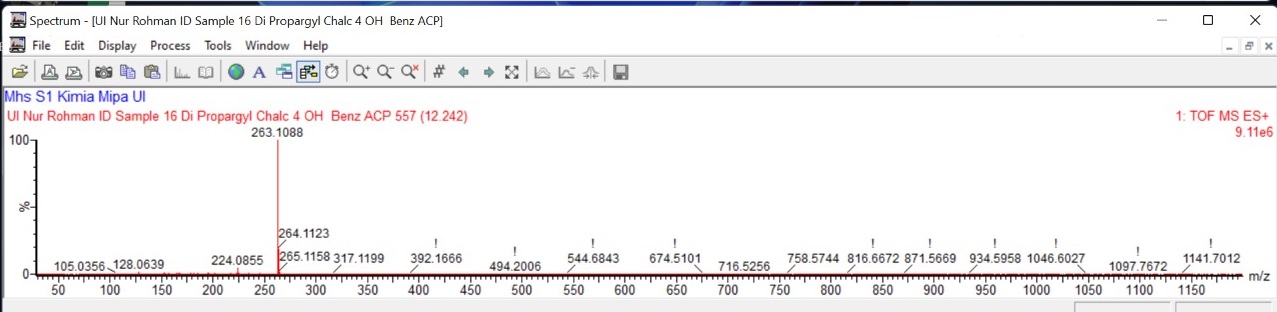


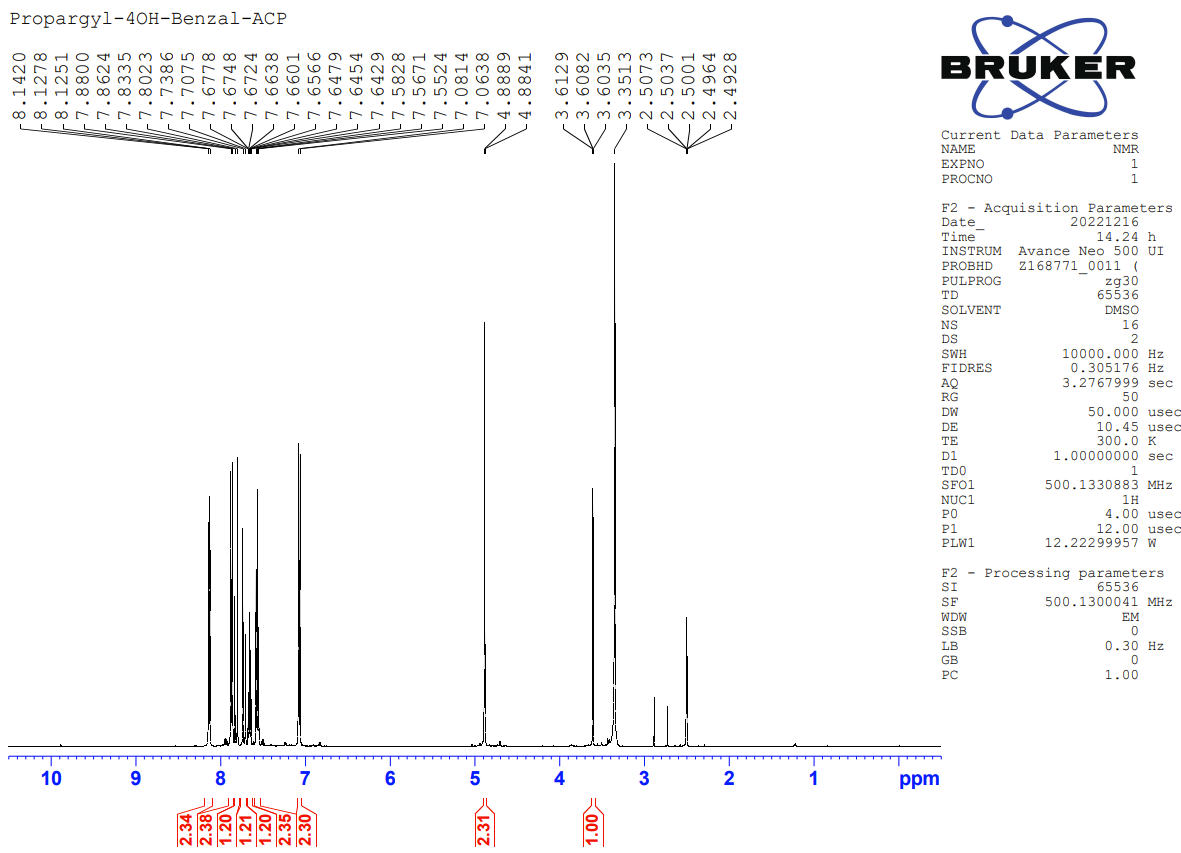


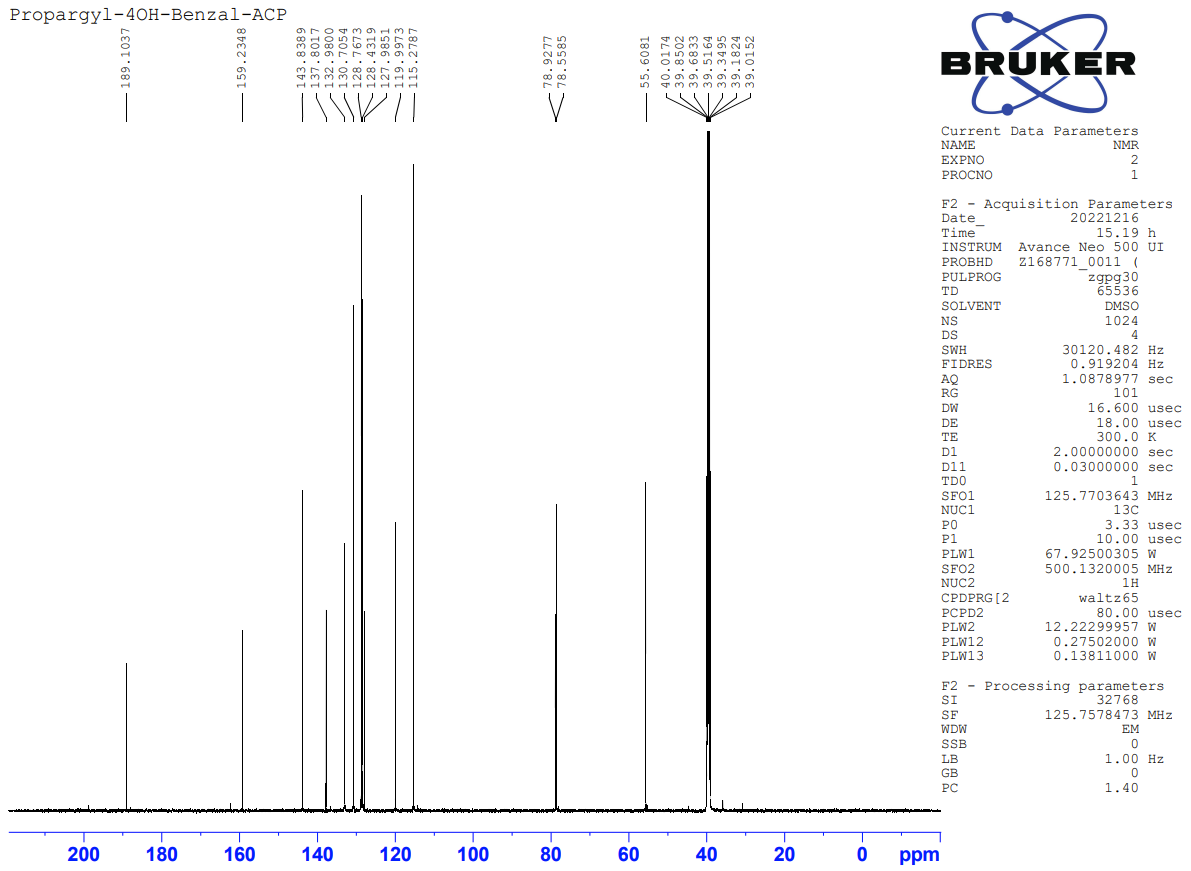


***(E)-3-(3-methoxy-4-(prop-2-yn-1-yloxy)phenyl)-1-phenylprop-2-en-1-one (3b):***

Off-white solid. R*_f_* value 0.80 (hexane / ethyl acetate = 3 / 1); mp. 51–53 °C; IR (KBr, disc) ν_max_ 3296, 3249, 3074, 2970, 2128, 1655, 1589, 1507, 1451, 1266, 1140, 1015 cm^-1^; ^1^H NMR (500 MHz, CDCl_3_) δ 7.99 (dd, 2H, *J* = 7.8, 1.4 Hz), 7.74 (d, 1H, *J* = 15.7 Hz), 7.69 (dd, 1H, *J* = 5.7, 3.4 Hz), 7.55 (t, 1H, *J* = 7.4 Hz), 7.50 (dd, 1H, *J* = 5.7, 3.4 Hz), 7.40 (d, 1H, *J* = 15.7 Hz), 7.21 (dd, 1H, *J* = 8.4, 1.9 Hz), 7.16 (d, 1H, *J* = 1.9 Hz), 7.03 (d, 1H, *J* = 8.3 Hz), 4.79 (d, 2H, *J* = 2.4 Hz), 3.91 (s, 3H), 2.54 (t, 1H, 2.4 Hz); ^13^C NMR (126 MHz, CDCl_3_) δ 190.4, 149.7, 149.0, 144.6, 138.3, 132.5, 130.8, 128.5, 128.3, 122.5, 120.4, 113.6, 110.6, 77.9, 76.2, 56.5, 55.9; HRMS (ESI-TOF) calcd for C_19_H_20_O_3_ [M+H]^+^ 293.1178, found 293.1197.


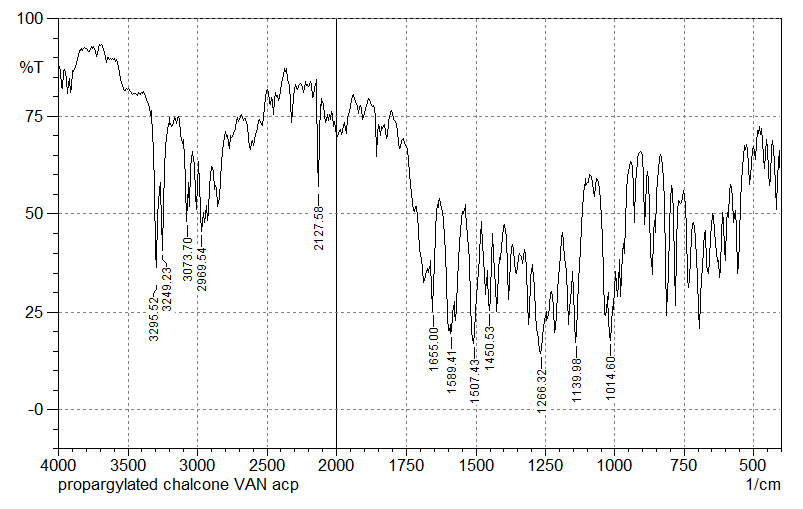


**
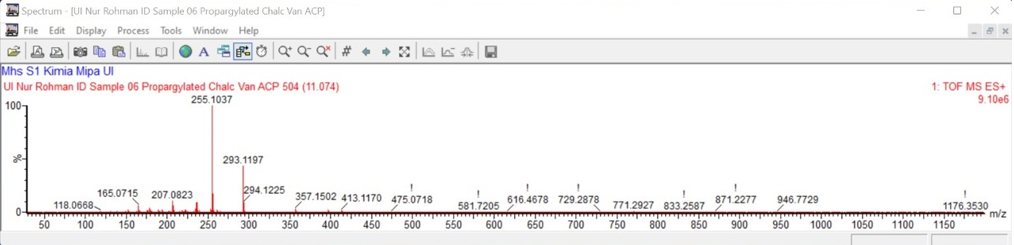
**


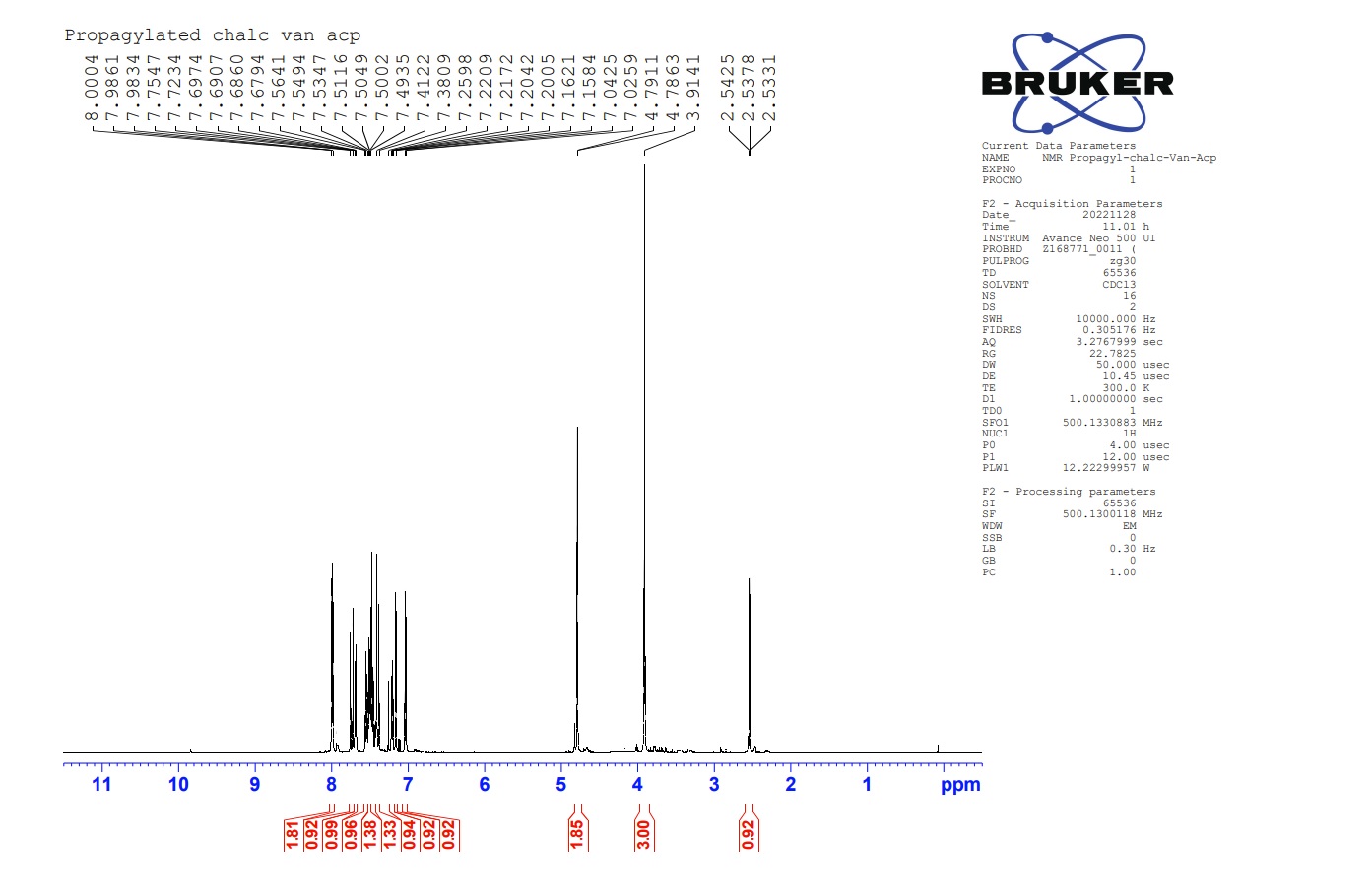


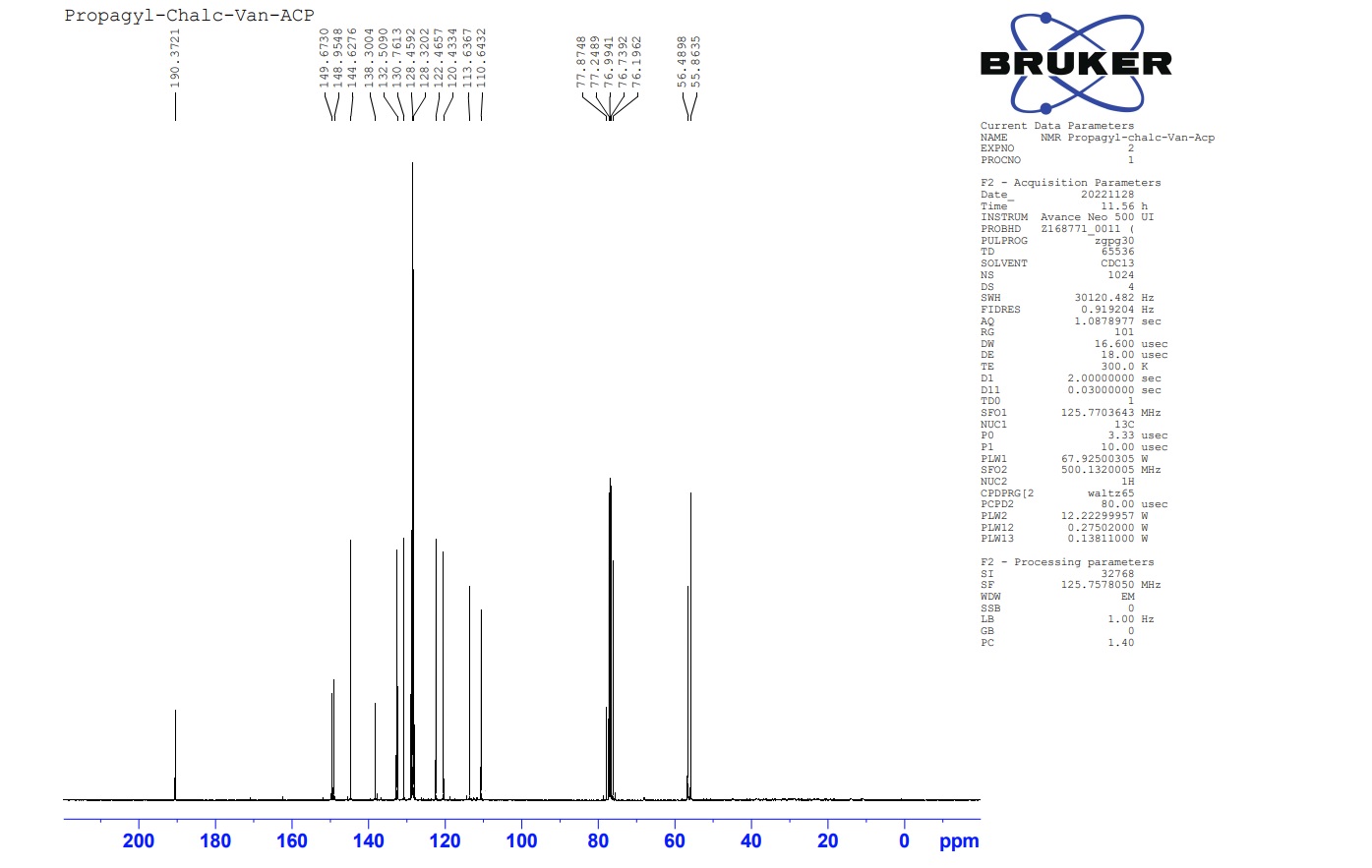


***(E)-3-(4-((1-(4-nitrophenyl)-1H-1,2,3-triazol-4-yl)methoxy)phenyl)-1-phenylprop-2-en-1-one (4ax):***

Yellow solid. R*_f_* value 0.40 (hexane / ethyl acetate = 1 / 1); m.p. 213–215 °C; IR (KBr, disc) ν_max_ 3094, 1654, 1586, 1525, 1507, 1340, 1248, 1215, 1172, 1022 cm^-1^; ^1^H NMR (500 MHz, DMSO-*d*6) δ 9.19 (s, 1H), 8.46 (s, 2H), 8.26 (s, 2H), 8.14 (s, 2H), 7.89–7.81 (m, 2H), 7.75–7.57 (m, 2H), 7.36 (s, 3H), 7.19 (s, 2H), 5.37 (s, 2H); ^13^C NMR (126 MHz, DMSO-*d*6) δ 189.1, 160.0, 146.7, 143.9, 140.8, 137.8, 133.0, 130.9, 128.8, 128.4, 126.4, 125.6, 123.5, 120.8, 120.1, 119.9, 115.2, 61.1; HRMS (ESI-TOF) calcd for C_24_H_19_N_4_O_4_ [M+H]^+^ 427.1406, found 427.1399.


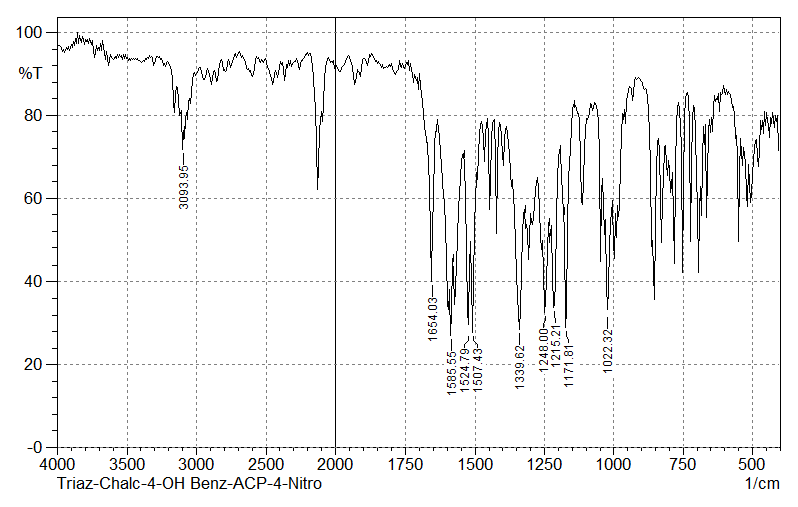


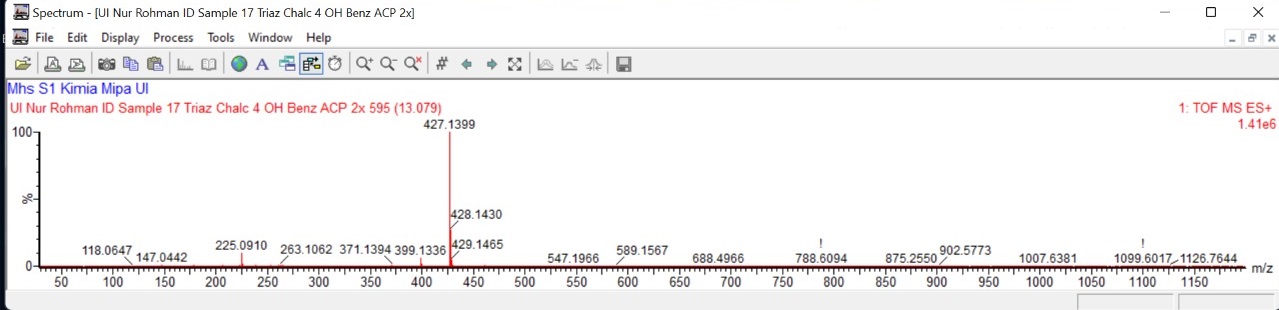


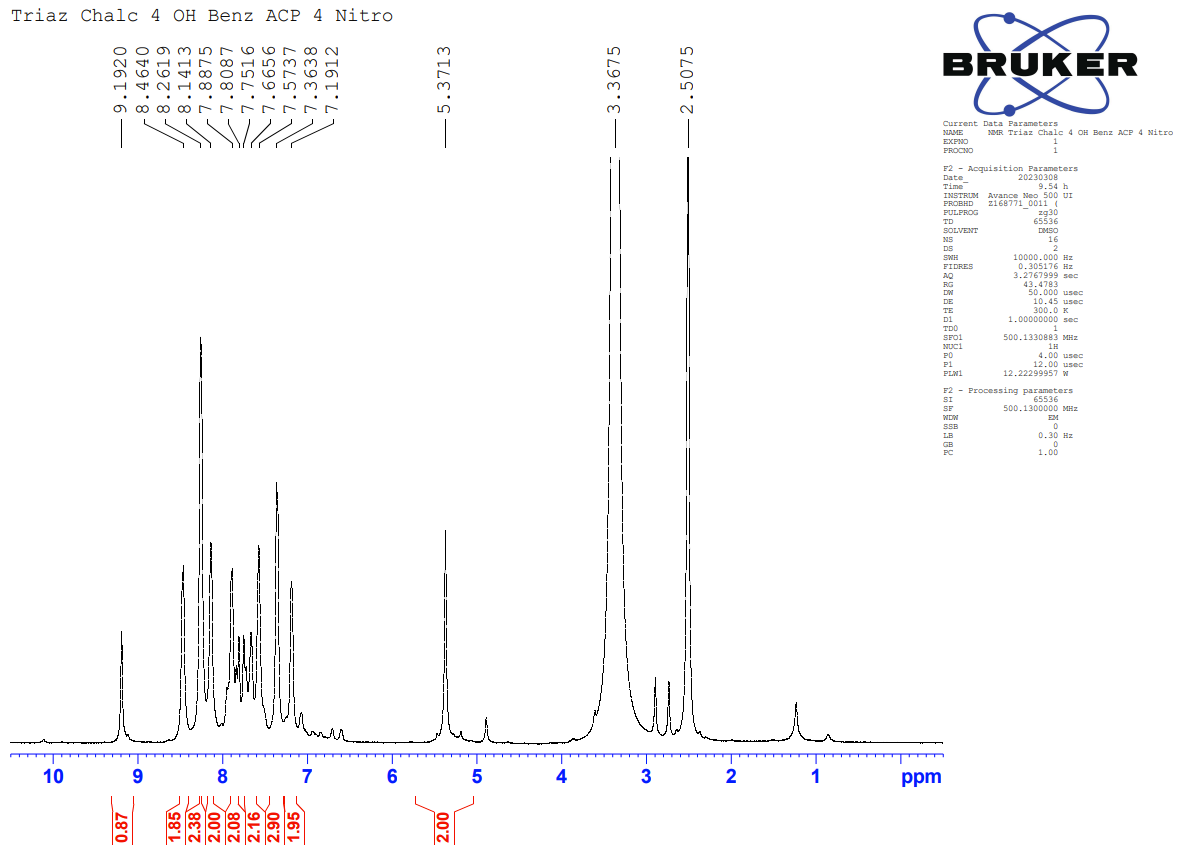


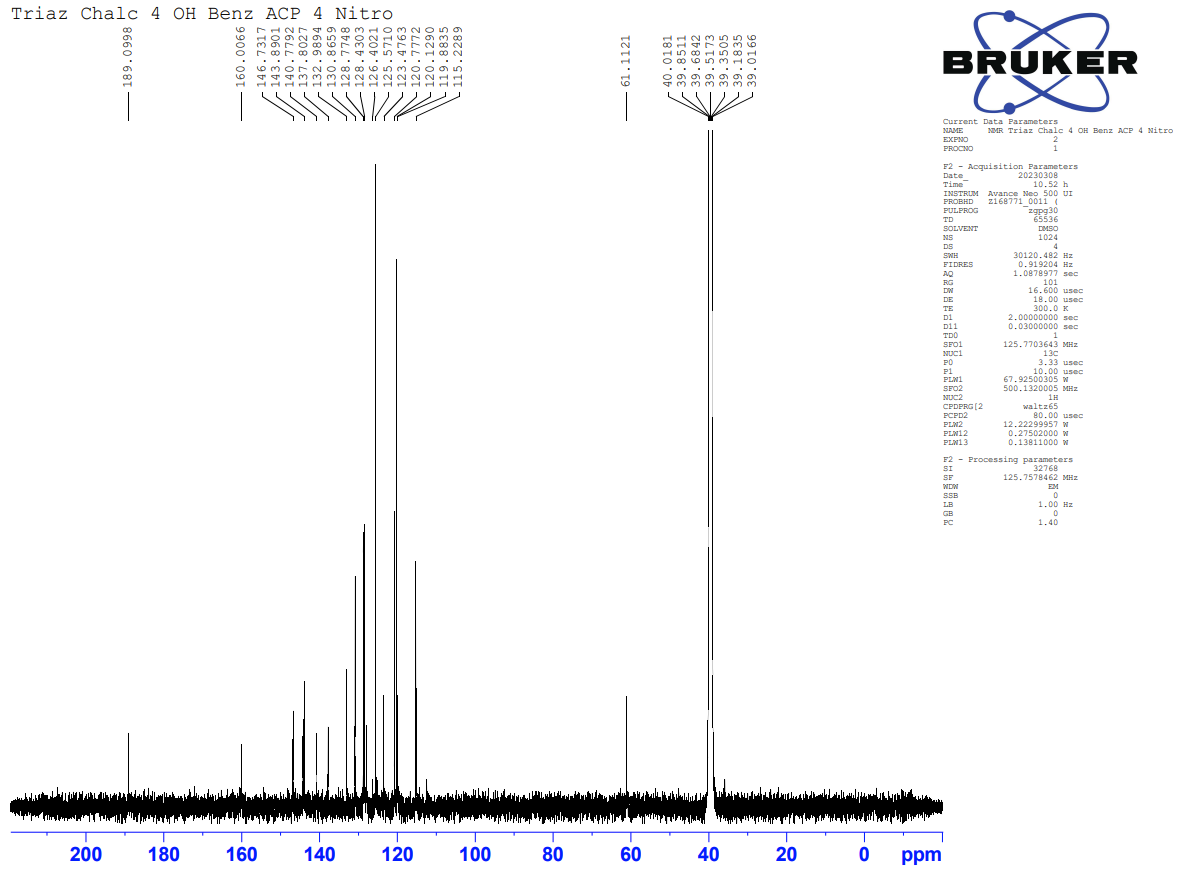


***(E)-3-(4-((1-(4-acetylphenyl)-1H-1,2,3-triazol-4-yl)methoxy)phenyl)-1-phenylprop-2-en-1-one (4ay):***

Yellow solid. R*_f_* value 0.88 (hexane / ethyl acetate = 1 / 1); mp. 199–200 °C; IR (KBr, disc) ν_max_ 3144, 1679, 1652, 1590, 1514, 1259, 1219, 1177, 1037, 1019, 986 cm^-1^; ^1^H NMR (500 MHz, DMSO-*d*6) δ 9.13 (s, 1H), 8.19 (d, 2H, *J* = 8.5 Hz), 8.14 (d, 2H, *J* = 7.9 Hz), 8.11 (d, 2H, *J* = 8.0 Hz), 7.90 (d, 2H, *J* = 8.5 Hz), 7.83 (d, 1H, *J* = 15.5 Hz), 7.74 (d, 1H, *J* = 15.5 Hz), 7.67 (t, 1H, *J* = 7.4 Hz), 7.58 (t, 2H, *J* = 7.4 Hz), 7.19 (d, 2H, *J* = 8.2 Hz), 5.36 (s, 2H), 2.65 (s, 3H); ^13^C NMR (126 MHz, DMSO-*d*6) δ 197.0, 189.1, 160.1, 143.98, 143.91, 139.5, 137.8, 136.5, 133.0, 130.9, 130.1, 128.9, 128.4, 127.8, 123.2, 119.94, 119.87, 115.3, 61.2, 26.9; HRMS (ESI-TOF) calcd for C_26_H_22_N_3_O_3_ [M+H]^+^ 424.1661, found 424.1653.


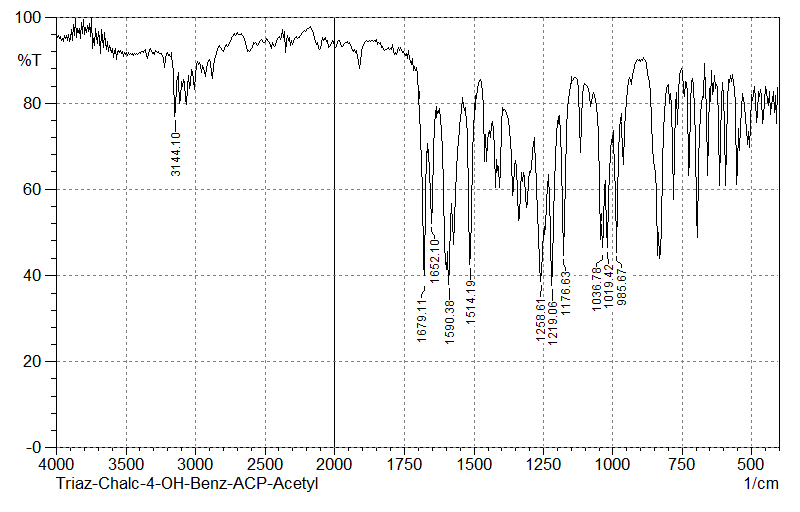


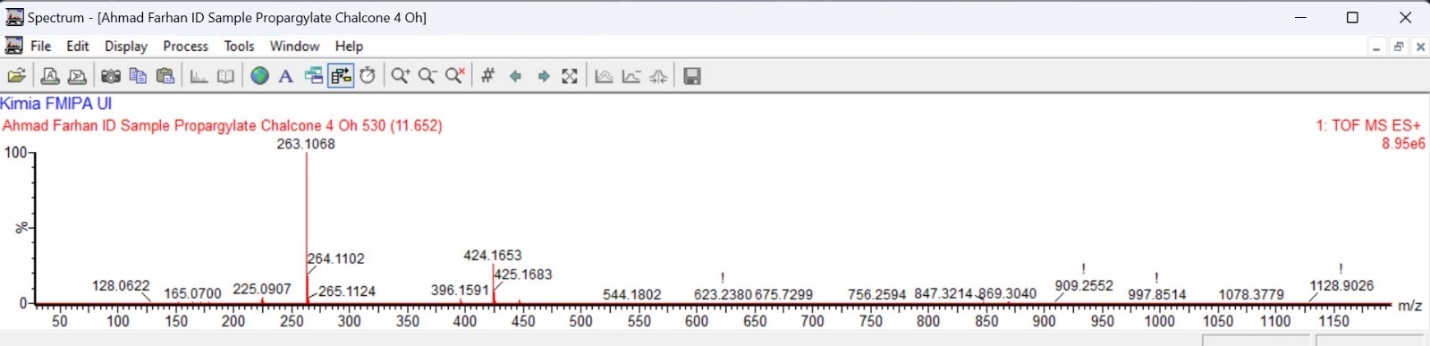


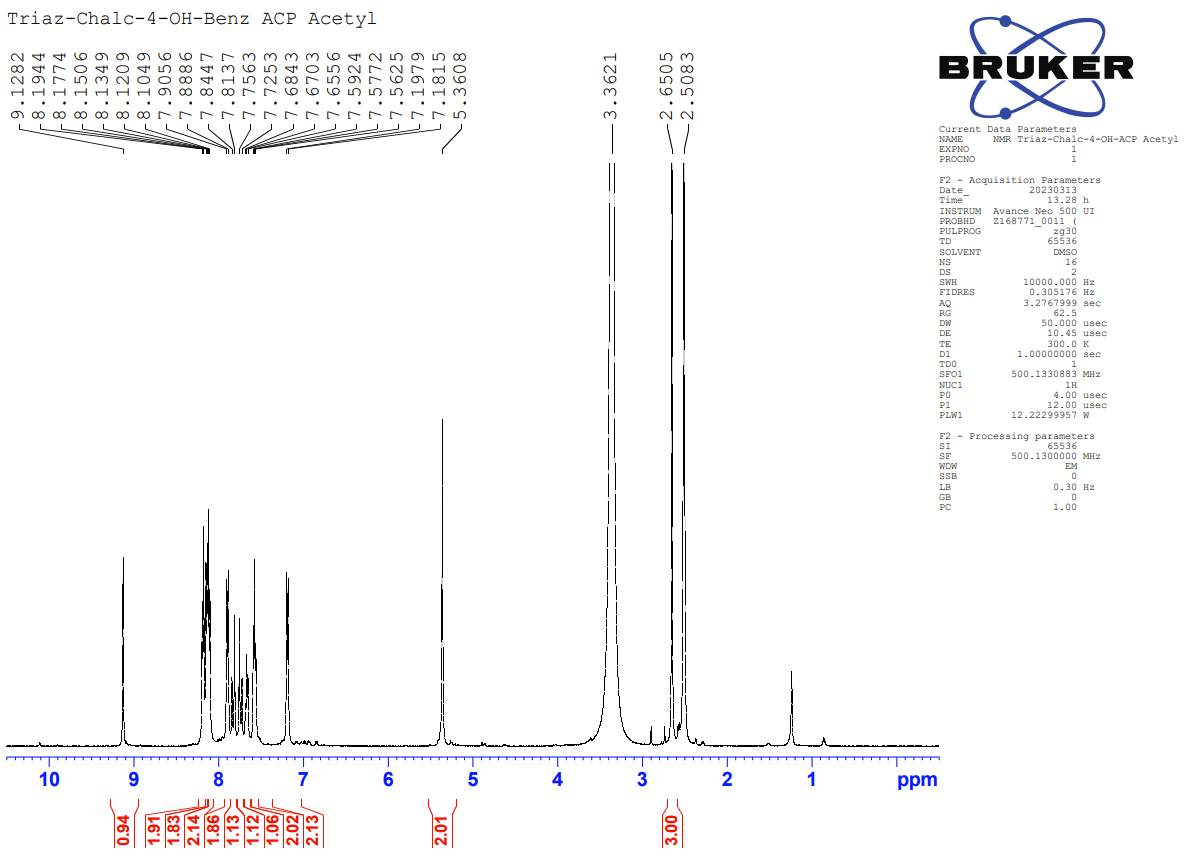


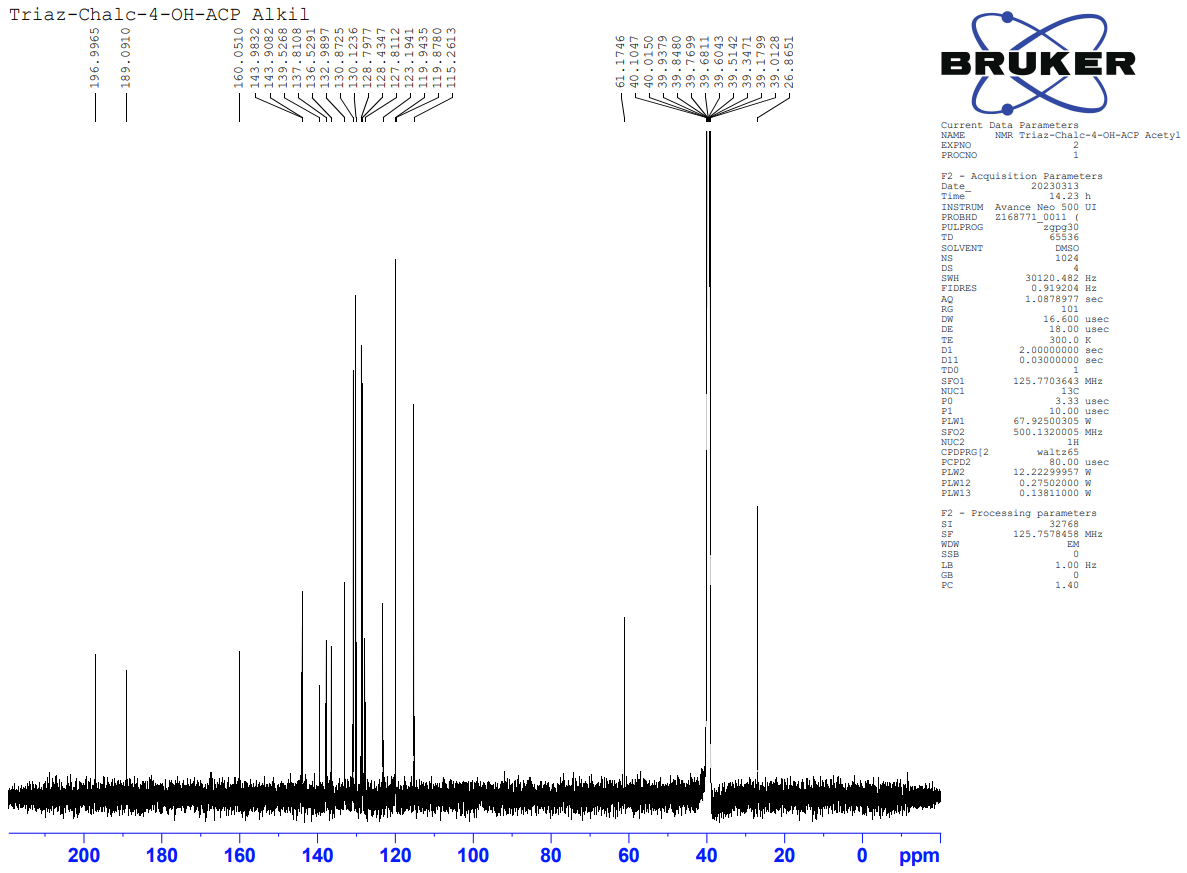


***(E)-3-(3-methoxy-4-((1-(4-nitrophenyl)-1H-1,2,3-triazol-4-yl)methoxy)phenyl)-1-phenylprop-2-en-1-one (4bx):***

Orange solid. R*_f_* value 0.30 (hexane / ethyl acetate = 3 / 1); mp. 197–199 °C; IR (KBr, disc) ν_max_ 3142, 3094, 2960, 2873, 1728, 1653, 1593, 1513, 1343, 1260, 1139 cm^-1^; ^1^H NMR (500 MHz, DMSO-*d*6) δ 9.18 (s, 1H), 8.46 (d, 2H, *J* = 9.1 Hz), 8.25 (d, 2H, *J* = 9.1 Hz), 8.15 (d, 2H, *J* = 7.3 Hz), 7.84 (d, 1H, *J* = 15.6 Hz), 7.72 (d, 1H, *J* = 15.8 Hz), 7.68–7.66 (m, 1H), 7.57 (t, 3H, *J* = 7.8 Hz), 7.42 (dd, 1H, *J* = 8.2, 1.5 Hz), 7.28 (d, 1H, *J* = 8.4 Hz), 5.33 (s, 2H), 3.86 (s, 3H); ^13^C NMR (126 MHz, DMSO-*d*6) δ 189.0, 149.7, 149.2, 146.8, 144.3, 144.1, 140.7, 137.8, 132.9, 128.7, 128.6, 125.5, 123.7, 123.1, 120.7, 119.9, 113.3, 111.1, 67.4, 55.7; HRMS (ESI-TOF) calcd for C_25_H_21_N_4_O_5_ [M+H]^+^ 457.1512, found 457.1523.


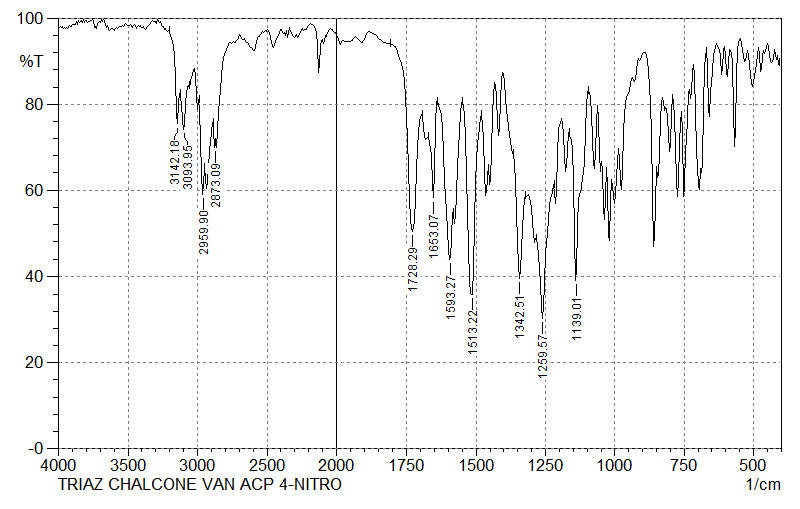


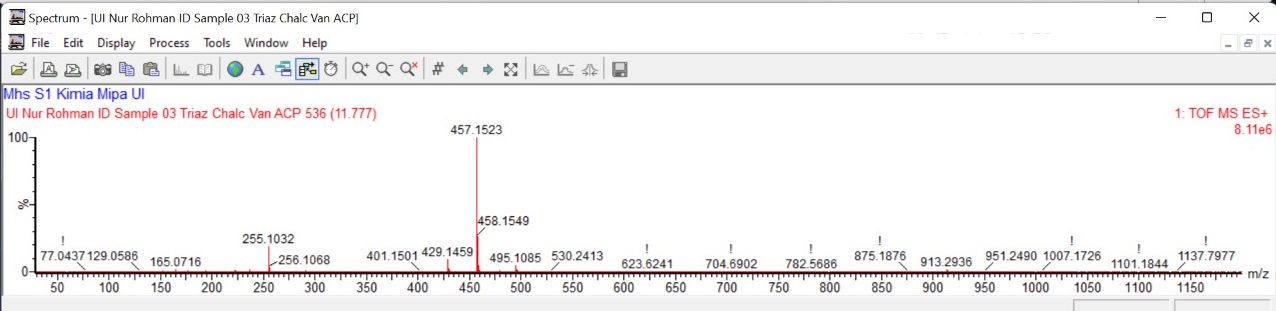


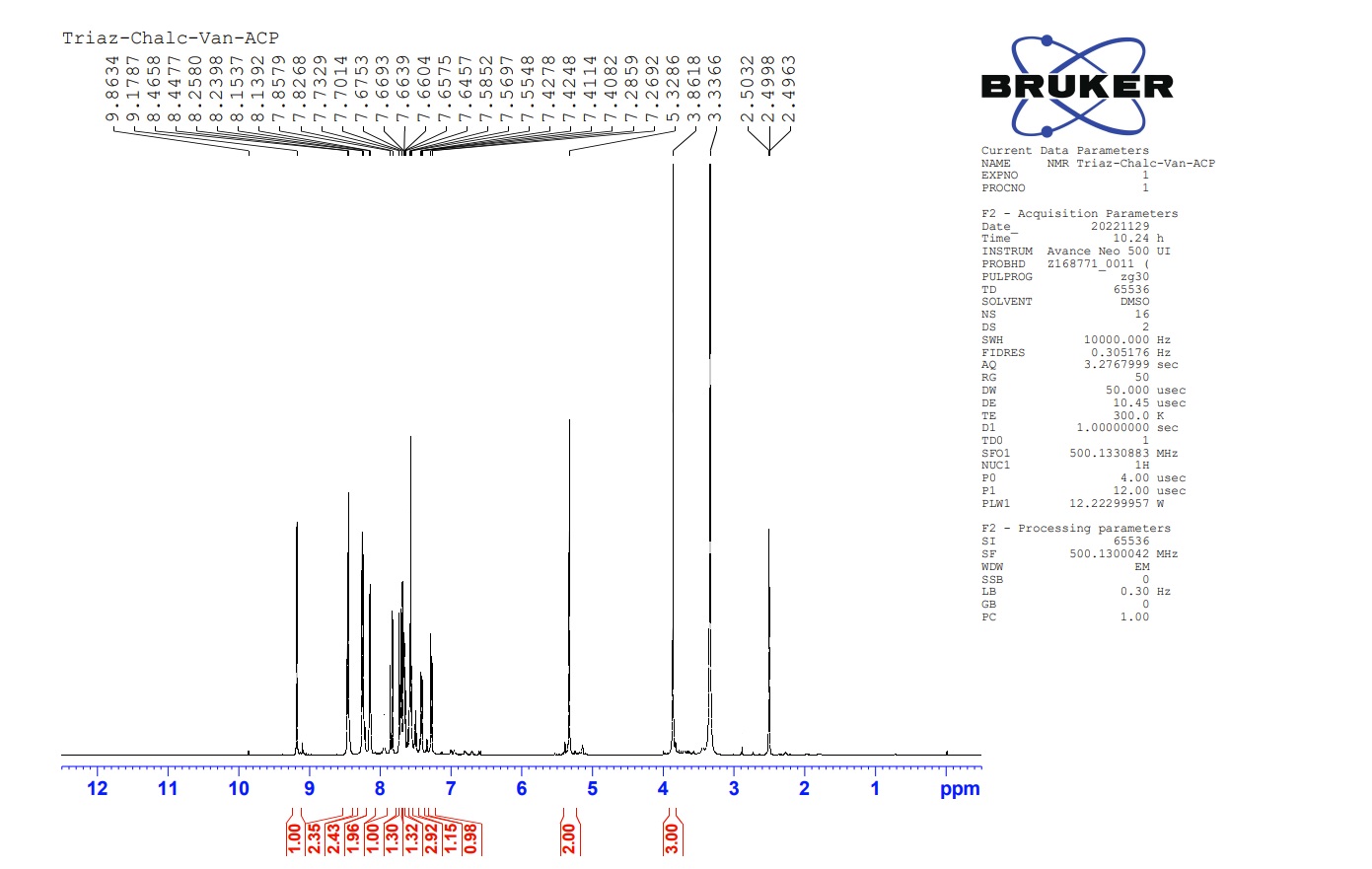


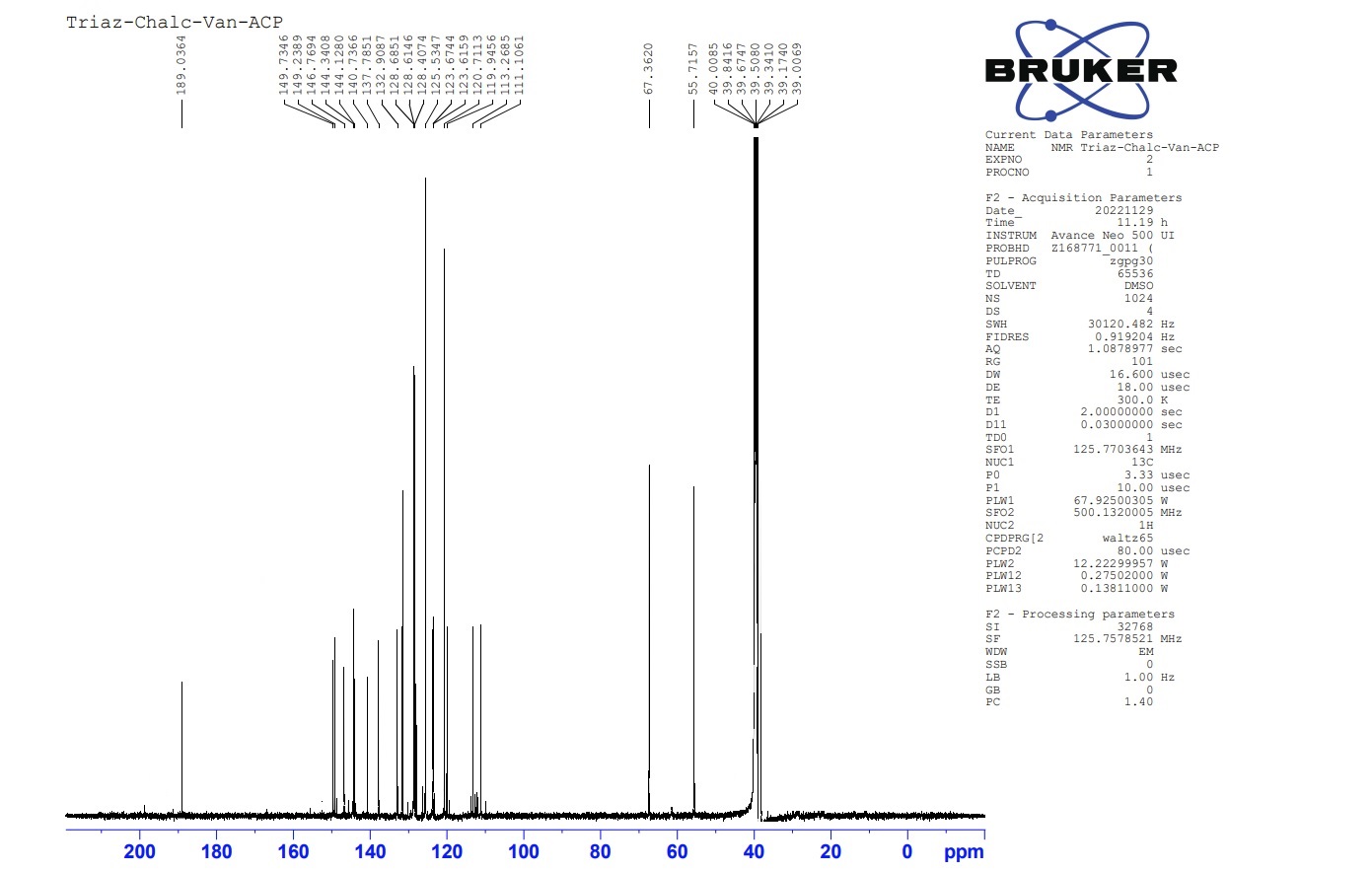


***(E)-3-(4-((1-(4-acetylphenyl)-1H-1,2,3-triazol-4-yl)methoxy)-3-methoxyphenyl)-1-phenylprop-2-en-1-one (4by):***

Orange solid. R*_f_* value 0.85 (hexane / ethyl acetate = 1 / 1); mp. 104–106 °C; IR (KBr, disc) ν_max_ 3138, 2952, 1681, 1651, 1605, 1513, 1260, 1137, 1018 cm^-1^; ^1^H NMR (500 MHz, DMSO-*d*6) δ 9.12 (s, 1H), 8.18 (d, 2H, *J* = 8.6 Hz), 8.15 (d, 2H, *J* = 7.8 Hz), 8.11 (dd, 2H, *J* = 8.8, 2.5 Hz), 7.85 (d, 1H, *J* = 15.5 Hz), 7.71 (d, 1H, *J* = 15.6 Hz), 7.60–7.57 (m, 4H), 7.46–7.40 (m, 2H), 3.88 (s, 3H), 3.83 (s, 2H), 2.65 (s, 3H); ^13^C NMR (126 MHz, DMSO-*d*6) δ 197.0, 189.1, 149.4, 148.9, 144.3, 143.5, 139.5, 137.8, 136.5, 133.0, 128.8, 128.5, 125.8, 123.4, 120.1, 120.0, 113.5, 112.8, 111.2, 109.8, 78.6, 55.5, 26.9; HRMS (ESI-TOF) calcd for C_27_H_23_N_3_O_4_ [M+H]^+^ 454.1767, found 454.1760.


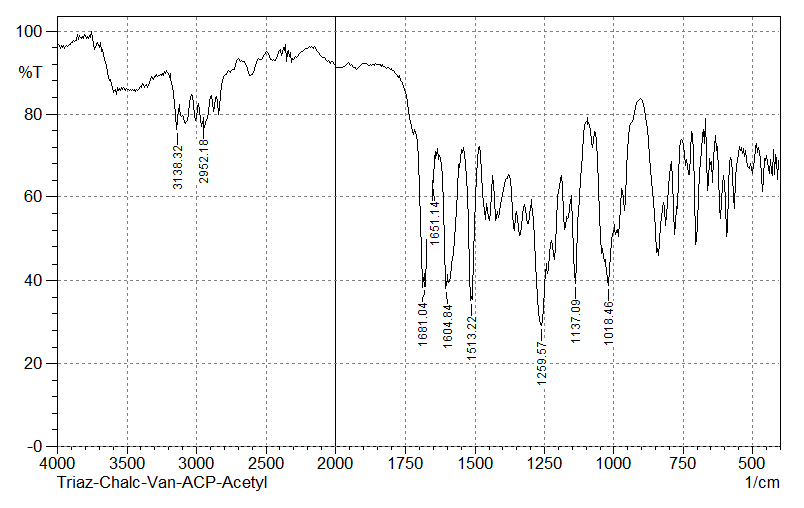


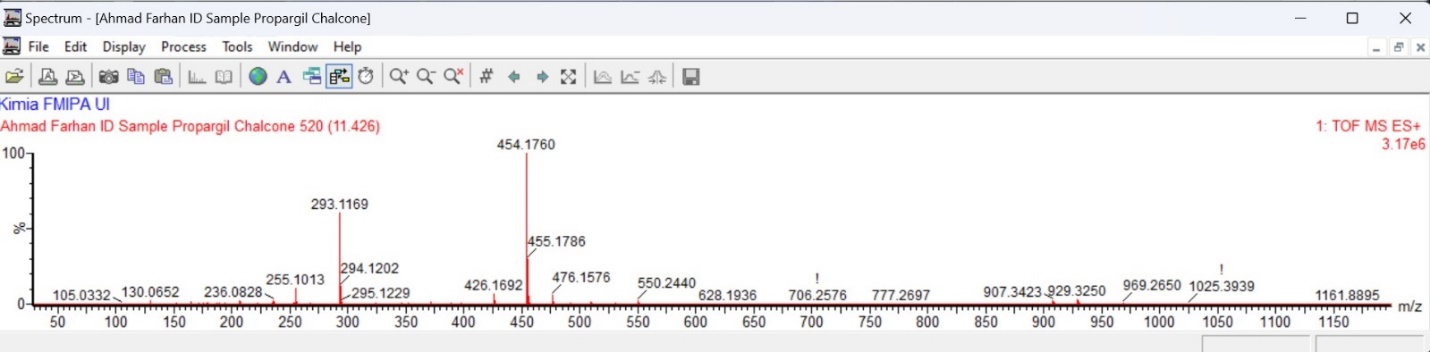


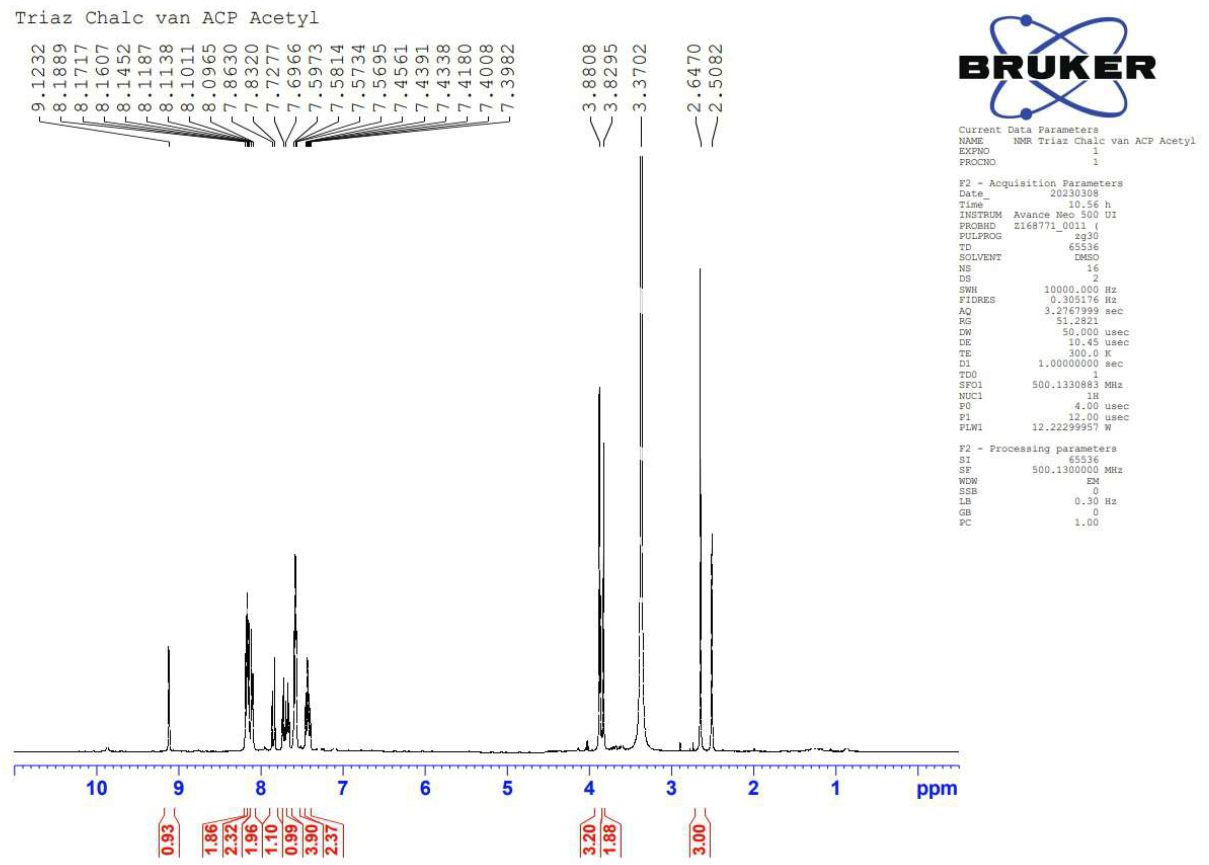


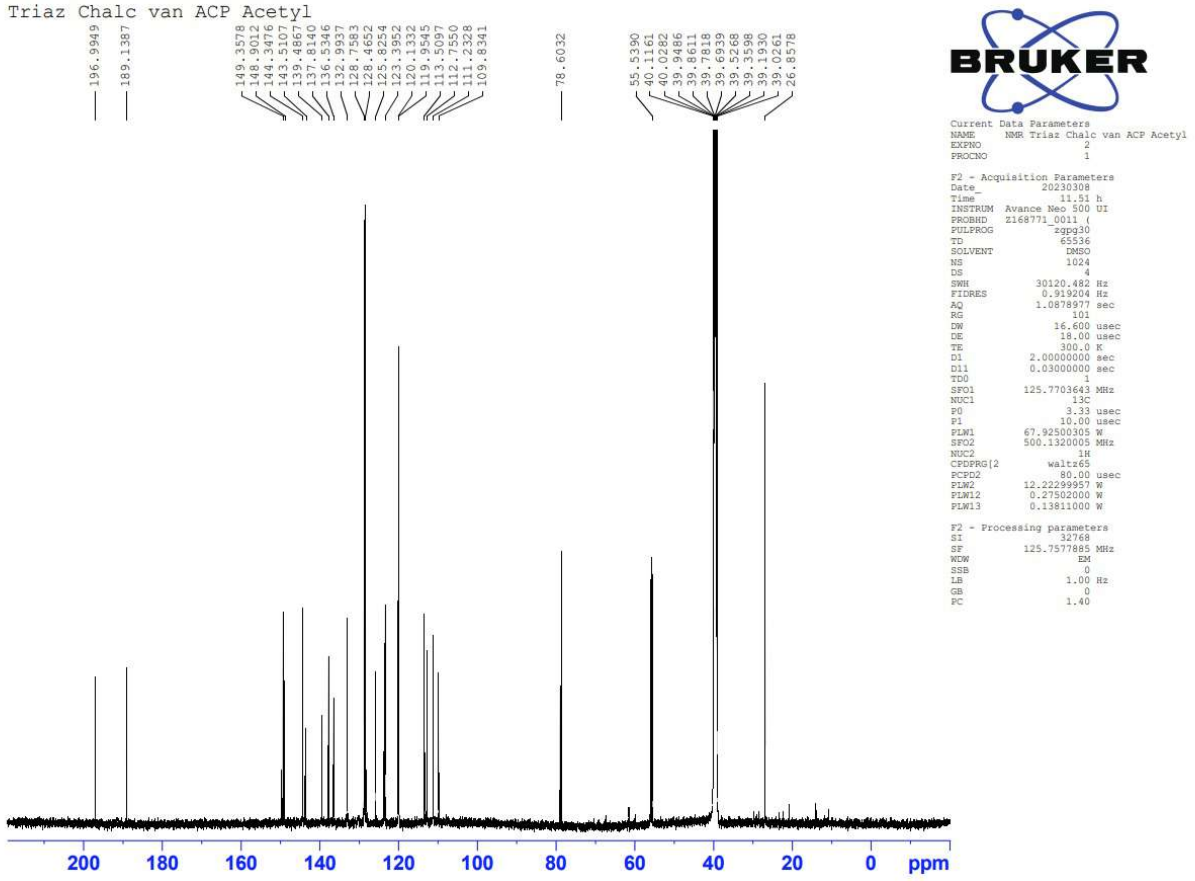

Supplement: Supplementary file 1 [file mmc1.docx]
